# Cognitive Reserve Characteristics and Occupational Performance Implications in People with Mild Cognitive Impairment

**DOI:** 10.3390/healthcare9101266

**Published:** 2021-09-26

**Authors:** Cristina Mendoza-Holgado, Jesús Lavado-García, Fidel López-Espuela, Raúl Roncero-Martín, María Luz Canal-Macías, Vicente Vera, Ignacio Aliaga, Purificación Rey-Sánchez, Juan Diego Pedrera-Zamorano, Jose M. Moran

**Affiliations:** 1 Occupational Therapist in Health and Social Services Department, Government of Extremadura, 10001 Cáceres, Spain; cristina.mendoza@salud-juntaex.es; 2 Metabolic Bone Diseases Research Group, Nursing Department, University of Extremadura, 10003 Cáceres, Spain; jmlavado@unex.es (J.L.-G.); rronmar@unex.es (R.R.-M.); luzcanal@unex.es (M.L.C.-M.); i.aliaga@pdi.ucm.es (I.A.); prey@unex.es (P.R.-S.); jpedrera@unex.es (J.D.P.-Z.); jmmorang@unex.es (J.M.M.); 3 Department of Stomatology II, School of Dentistry, Complutense University, 28040 Madrid, Spain; vicentevera@odon.ucm.es

**Keywords:** mild cognitive impairment, dementia, cognitive reserve, occupational therapy

## Abstract

The Cognitive Reserve hypothesis suggests that there are individual differences in the ability to cope with the pathologic changes in Alzheimer’s Disease. The proportion of elderly individuals has increased in recent years; this increase emphasizes the importance of early detection of mild cognitive impairment and the promotion of healthy ageing. The purpose of our study is to characterize cognitive reserve and occupational performance implications in people with mild cognitive impairment. 125 patients with mild cognitive impairment were enrolled. The Montreal Cognitive Assessments (MoCA) was used to evaluate cognitive status and the Cognitive Reserve Index Questionnaire (CRIq) as an indicator of cognitive reserve. Higher level of education was associated with higher MoCA scores (r = 0.290, *p* = 0.001). Positive significant correlations were observed between MoCA and total CRIq (r = 0.385, *p* < 0.001) as well as its three sub-domains, education (r = 0.231, *p* = 0.010), working activity (r = 0.237, *p* = 0.008) and leisure time (r = 0.319, *p* < 0.001). This study findings provide the importance of considering socio-behavioral factors in cognitive status. This research helps to describe the importance of engaging occupationally along the whole life-course as a potential protective factor in ageing, and includes a perspective of occupational therapy regarding the hypothesis of cognitive reserve.

## 1. Introduction

In recent years life expectancy has increased worldwide, and so has the prevalence of patients with dementia and cognitive impairment. An estimated 46.8 million people worldwide are suspected to have dementia [1]. In Spain, the proportion of elderly individuals has increased to 19.6%. By 2050, the proportion of elderly is estimated to increase to 31.4% [2]. This rise emphasizes the importance of early detection and treatment of dementia [3]. Many clinical efforts have been made to understand mild cognitive impairment and neurodegenerative disorders. Because of the aging population there is an increasing interest in studying the aging brain and potential protective factors, and a growing interest in the pre-dementia phase [4,5].

Mild Cognitive Impairment (MCI) represents a transitional state between healthy aging and very mild Alzheimer’s Disease (AD), involving a cognitive decline which is greater than expected for that age [5]. This clinical concept has generated a considerable amount of interest during recent years in relation to its significance as a possible prodromal phase of AD. The Montreal Cognitive Assessment (MoCA) is a cognitive screening test designed to assist health professionals in the detection of mild cognitive impairment, a tool used as a quick screening test which has shown excellent sensitivity in identifying MCI and AD [3,6].

The cognitive reserve (CR) hypothesis is an active model that suggests that there are individual differences in the ability to cope with the pathologic changes in AD [7]. Although this definition is suitable for both healthy individuals and those with brain damage, the concept of CR has found potential applications particularly in the field of brain pathologies [8]. Aspects of life experience may supply reserves in the form of a set of skills that allow some people to cope with the pathologic changes better than others. Epidemiologic and functional imaging studies have also provided support for the concept of CR [8,9,10].

**Figure 1:**
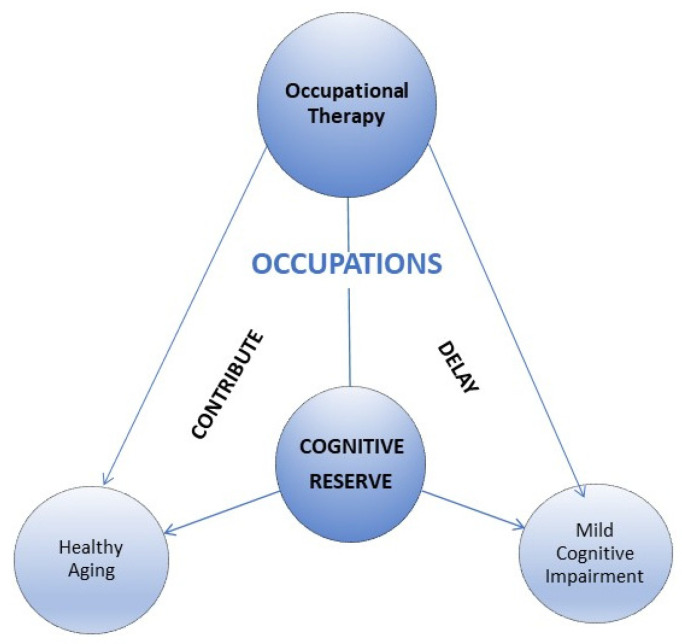
Interaction between occupational therapy and cognitive reserve.

The CR model is in line with one of most common statements in Occupational Therapy, i.e., that adequate balance in the activities of daily living is essential for health and well-being [11,12]. Moreover, meaningful and purposeful activities, or occupations, have a stronger effect on health [13]. The CR model suggests that a person’s lifestyle and occupations will determine his or her CR (Figure 1). Socially active people who have engaged in leisure activities, usually classified into intellectual activities such as reading books and learning languages or productive activities such as knitting and gardening, and who have had cognitively demanding jobs, will have an increased CR that will allow them to cope more effectively with cognitive decline associated with ageing or dementia [14].

On the other hand, one of the primary impacts of an illness or injury is the cessation of the ability to engage in personally meaningful occupations [15]. However, occupational therapists should be concerned with human well-being rather than being preoccupied solely with people who have impairments [16].

A great deal of interest has been generated concerning CR. Educational and occupational complexity attainments are considered such that aspects of life experience have been extensively investigated, and studies have shown that patients with higher educational or occupational complexity attainments deal better with brain damage [17,18]. However, exploration of the influence of a socially and intellectually engaged lifestyle on dementia has not been considered until recently, studies suggesting that engaging in various social, intellectual and leisure activities reduces the risk of prevalence or incidence of AD before it is expressed clinically [19,20,21,22]. These variables have been commonly used as proxies for CR. These factors are usually interrelated with environmental factors [23]. Other complementary concepts have emerged around CR, such as brain reserve or brain maintenance [24].

Moving towards an identification of CR due to these implications as protective factors is a necessity; nevertheless, there has been confusion regarding the measurement of these constructs and the appropriate ways to apply them to research proxies for CR [25]. Several measures assessing CR have been developed [26]. The Cognitive Reserve Index questionnaire (CRIq) was proposed by Nucci and its principal advantage is that it measures three subdomains (education, working activity and leisure time), according to the theoretical statement of the CR construct [3,27].

Moreover, other factors are considered as risk factors and could contribute to cognitive decline. Depression mood disorder commonly occurs in association with mild cognitive impairment, and evidence suggests that depression confers a higher rate of progression along the neurodegenerative spectrum from MCI to dementia [28,29]. Evaluation of depressive syndromes in cognitively impaired patients is complicated by the symptom overlap with dementia [30], but symptoms of depression in geriatric patients have been detected using the Spanish version of the 15-point Geriatric Depression Scale [31].

The interrelation between these ideas is complex. Further, differences have been found between elderly who are considered cognitively healthy and those with MCI. Literature reviews have reported that there is a paradoxical relationship between CR and clinical progression [32]. Patients with AD and higher educational and occupational attainment have more rapid decline than those with lower attainment, consistent with the theory that the underlying AD pathology is more advanced in patients with more CR in contrast with normal aging [9,32]. Previously research has found that MoCA reflects CR in healthy people over 60 years old [3], but this does not report if the association still persists in people with cognitive impairment.

Furthermore, it is important to develop research into the relationship between mild cognitive impairment and cognitive reserve due its implication in the development of and possible progression to dementia. Therefore, here we present an emerging field of research in which it is hypothesized that the occupations performed in a lifespan have implications on the way people age, and can affect functionality.

## 2. Materials and Methods

A cross-sectional study was carried out aiming to investigate CR characteristics and occupational performance implications in people with MCI. In addition, we consider whether there is a paradoxical relationship between CR and MCI. 

### 2.1. Participants

A total of 125 participants aged 60–90 years with a mean age of 74.26 ± 6.64 years, all community-dwelling people with MCI, were recruited from February to March 2020 from Occupational Therapy consultations framed in the Plan Integral de Atención al Deterioro Cognitivo de Extremadura(PIDEX) developed by the Consejería de Sanidad y Políticas Sociales de Extremadura (Spain). All participants had previously undergone clinical screening by a neurologist and neuropsychologist which determined a formal diagnosis of Cognitive Decline or subjective memory complaints.

All participants met the inclusion criteria as follows: (1) a formal diagnosis of mild cognitive impairment, (2) Global Deterioration Scale score between 3 and 4, (3) community-dwelling, (4) 60 years and over. Exclusion criteria were as follows: (1) psychiatric disorders, (2) presence of sensorial impairments that would hinder the assessment, (3) moderate-severe anosognosia, (4) cognitive impairment relative to aphasia (comprehensive or expression).

### 2.2. Ethical Considerations

The Clinical Research Ethics Committee approved this study (Code -012-2020). All participants provided written informed consent; the study was performed in accordance with the 1975 Declaration of Helsinki. Before the study began, the main researcher explained the objective, study methodology, data collection method, expected benefits and the inconveniences caused by participation in this study.

### 2.3. Study Tools

To collect sociodemographic characteristics of the subjects (age, gender, level of education, marital status, living status, etc.), information about medical history and level of Global Deterioration Scale (GDS), which is a brief clinical rating of dementia severity [33], a general interview and questionnaire was developed by the main researcher.

Cognitive status was assessed with the Spanish version of The Montreal Cognitive Assessment [MoCA] [6]. This is a screening instrument used to detect mild cognitive impairment by assessing various domains of cognition.

Higher scores indicate better cognition; the maximum score is 30. Subjects who achieve scores less than 26 are suspected of MCI. This test involves several tasks and categories of cognitive function (visuospatial/executive category, short term memory and recall memory, language category, attention, naming, abstraction and orientation to time and space).

To value the CR, CRIq was implemented. The CRIq evaluates the CR of an individual compilating information relating to the entire adult life. The questionnaire is divided into three sections: CRI-Education, CRI-WorkingActivity, CRI-LeisureTime. These sections are based on the theoretical construct of CR proposed by Stern [7]. Education (CRI-Education) is used to consider the years of formal and non-formal education. Occupation (CRI-WorkingActivity) measures the years of working and considers five categories with different degrees of responsibility and demands. Leisure and Social (CRI-Leisure) measures the value of different activities regarding their frequency and periodicity. The questionnaire was administrated to each participant and contrasted at the same time with a close relative, and was administered to their caregiver only when necessary due to memory impairment. Finally, the CR Index is the total score resulting from the combination of the sub-scores of each section adjusted by age; a higher aggregation means a higher level of CR. The short form of the Geriatric Depression Scale (GDS-15) is an instrument specifically designed to assess depression in geriatric populations. Its items require a yes/no response to 15 questions. A score of 0–5 indicated no depression; 6–9 suggested possible depression, and ≥10 revealed an established depression.

### 2.4. Statistical Analysis

The descriptive analysis was performed by calculating the percentage of categorical variables and the mean, together with the standard deviation in continuous variables. The Kolmorgorov-Smirnov test was used to check the normality of the variables if the data did not follow a normal distribution. The Mann-Whitney U test was used with continuous variables. To measure correlations between the demographic variables and MoCA score Spearman correlation and uni-variate linear regression analyses (independent variable: age, sex, and years of education) were used. The interactions between MoCA scores with CRIq as well as the three subdomains, education, working activity and leisure time, were analyzed by bivariate correlations. Analyses were run by SSPS (version 25, IBM, Armonk, NY, USA). Statistical significance was established when *p* < 0.05.

## 3. Results

A total of 125 older adults with MCI were enrolled. The sociodemographic and clinical characteristics of the patients are shown in Table 1. The mean age of the study participants was 74.3 years (SD ± 6.6 years). A total of 55 patients (44.0%) were 60–74 years and 70 (56.0%) were between 75–90 years old. A total of 28.0% of subjects were diagnosed with non-specific MCI and 28.0% suffered an amnesic MCI or probable AD. The majority of patients were women. Gender, age and geriatric depression scale score were not significantly different between groups. In contrast, MoCA (*p* < 0.016), CRI questionnaire total score (*p* < 0.001) and working-activity subdomain (*p* < 0.001), showed statistically significant differences, while the other subdomains, education and leisure time, were not statistically significant.

Table 2 shows the difference between MoCA scores based on demographic variables: age, sex and educational.

Based on within-group comparisons the male group showed higher MoCA scores than female (*p* = 0.016); higher level of education was associated also with higher MoCA score (correlation analysis r = 0.261, *p* = 0.001). Regarding the amount of difference between age groups, MoCA score did not find correlation (correlation analysis r = −0.139, *p* = 0.123) (Table 3).

**Figure 2:**
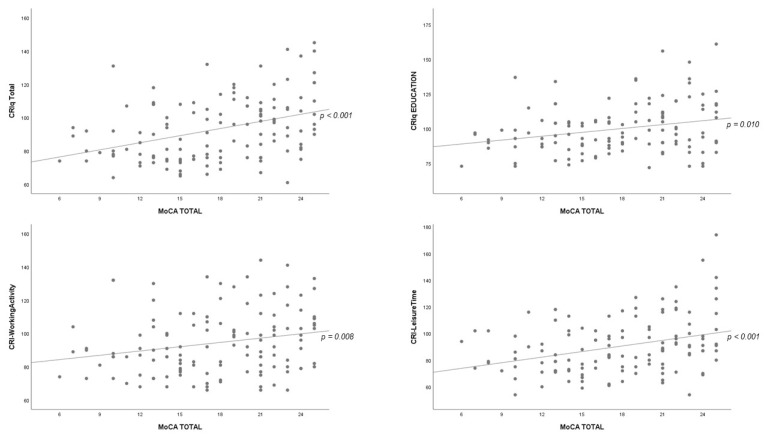
Scatter Plot for correlation analyses between CRIq score (Total and sub-domains) and MoCA score.

The results of analysis of bivariate correlations of MoCA scores with CRIq as the total score and the three subdomains are shown in Table 4. Positive significant correlations were observed with total CRIq (r = 0.385, 95% CI 0.21–0.53, *p* < 0.001); education (r = 0.231, 95% CI 0.06–0.39, *p* < 0.010); working activity (r = 0.237, 95% CI 0.06–0.40, *p* < 0.008) and leisure time (r = 0.319, 95% CI 0.15–0.47, *p* < 0.001) (Table 4) (Figure 2).

Confounding factors that could distort the associations detected between the variables of interest were taken into consideration by bivariate correlation analysis. In particular, sex, age and depression were controlled for, the correlations previously detected remaining mostly statistically significant and positive. Thus, correlations between MoCA Score and total CRIq Total score (r = 0.319, *p* < 0.001), as well as in the sub-domine CRI-Education (r = 0.254, *p* < 0.005) and CRI-LeisureTime (r = 0.298, *p* < 0.001) remained statistically significant, but not for CRI-WorkingActivity (r = 0.126, *p* = 0.165) (Table 5).

We further wanted to precisely determine the potential predictors of MoCA in this population by performing a multiple linear regression analysis with MoCA as dependent variable and CRIq and subdomains as independent. None of the factors included in the model served as a potential predictor of MoCA scores (*p* > 0.05) (Table 6).

## 4. Discussion

We aimed to investigate CR characteristics and occupational performance implications in people with MCI and the potential impact of a paradoxical relationship between CR and MCI. Moreover, we described the depressive status of patients.

Our study reflects that male subjects have better performance in MoCA, while the scores for females are lower with independence of age or educational trajectory. Again, for CRIq total score and subdomains (educational, working-activities, leisure time) females have the worse result reporting CRIq Total, working activity and leisure time lower scores. Furthermore, depressive symptoms were shown more often in women, although they did not represent a statistically significant difference.

The use of the cognitive reserve construct as a concept to prevent and anticipate the onset of dementia has increased in recent decades. Efforts have been made to find a good tool to characterize this specific condition, but structured assessments still have limitations [26]. In our study we use the Cognitive Reserve Index to estimate CR. This assessment has been reported in previous studies involving cognitive impairment. It is considered a suitable tool because it is short and easy to administer and it considers the three common socio-behavioral proxies included in the concept of cognitive reserve carried out throughout adulthood [7,25,27]. Nevertheless, other studies have used proxy measures which are not structured. Moreover, cognitive reserve and neuroplasticity theories recommend thorough research with neuroimaging data to explore the existence of possible compensatory neural mechanisms [8,25].

Regarding sex difference, we have observed better performance in males. This tendency was similar to that reported in previous studies [34,35] and significant sex differences also exist in the normal cognitive aging process [36,37]. Typically, studies have shown higher prevalence of AD in females than males and this has been attributed to their longer life spans [38]. In contrast, recent studies have considered more factors: sociocultural, neurobiology vulnerability, cultural customs, roles and access to education, etc. [35,39]. This complex vision of gender difference is in line with our results. Recently, another study has hypothesized that female gender is significantly associated with better functional ability [34]. One metanalysis underlines the importance of the etiology of cognitive impairment to understand sex difference in MCI, and reports no sex prevalence in amnestic MCI [40]. In addition, people with older age were more likely to progress to MCI because current knowledge suggests that advancing age is one of the greatest risk factors [39]. Therefore, in healthy aging, older people have worse results in cognitive assessment than younger [3]. However, these results were inconsistent with our findings that did not find correlation differences between groups of age sorted by gender. This inconsistency between our results and those in the published literature may be due to sample size or the cognitive profile of participants.

Many studies have reported on the comorbidity of depressive symptoms in MCI [41,42,43]. Depression is considered a modifiable risk factor, but the interaction with MCI is unclear. Some studies did not find a relationship between depressive symptoms and cognitive outcomes, and our results are similar to those published [44,45]. Stratifying by gender, we demonstrated that females have worse registers on a depressive scale, but this did not meet statistical significance. Women’s higher prevalence of depressive symptoms are broadly consistent with those reported in previous studies [36,46]. There is also evidence of an association between the severity of depressive symptoms and higher rate of conversion from MCI to dementia [36,47].

Our results were partially consistent with a study that showed how MoCA scores tended to be higher for patients with more years of education and younger age [3]. However, the relationship is poor, so our results potentially demonstrate that there is a paradoxical relationship between CR and MCI [32]. The effects of education as a protective factor to deal with neurodegenerative disorder have been widely reported in previous studies and our study reflects correlations between MoCA Score and education subdomain, working-activity and leisure time. However, previous research has shown that higher education was not associated with a slower rate of cognitive decline, so the contribution of CR may be limited [18].

Finally, our study supports the idea that occupational therapists need a critical approach to recognize the impact of inequities such a gender, education, etc., to acknowledge that well-being cannot be achieved by focusing solely on enhancing individuals’ abilities, and thus need to endeavor to facilitate change at both individual and environmental levels [13]. Engagement in occupations that provide purpose and meaning during the whole of life contribute to well-being and healthy aging, in particular, as a potential protective factor against cognitive decline [14]. Therefore, occupational therapists should work to support police and the law in avoiding occupational injustice in any area of daily activity, even in the healthy population [48,49].

### Limitations

Several limitations should be considered. First, our study did not check the clinical characterization of participants with structural image studies to contrast with the hypothesis of brain reserve. Secondly, although structured assessment is a suitable tool, bias risk could appear, such as loss of information regarding years of education, etc. Finally, our study takes into consideration a small sample selected by convenience, thus the result cannot be generalizable. In addition, more data about economic, functional status or clinical situation could be interesting to control as many factors as possible. Nevertheless, we highlight the importance of taking into consideration CR studies involving participants with mild cognitive impairment in order to have more information and apply better interventions to delay the onset of dementia.

## 5. Conclusions

In conclusion, this study’s findings show the importance of considering socio-behavioral factors in people with MCI and of occupational engagement during later life. Many aspects such as education, working activity and social and leisure time have an influence on cognitive status. The association of variable depression has not been directly addressed in this work. Here, we have used patient status related to depression as a covariate, a possible confounder that could modulate the associations of interest that have been studied.

Moreover, social demographics (gender) and occupational justice perspectives [49] have to be considered when we research cognition and MCI; our study demonstrates that female groups present worse presentation in cognitive status, depressive symptoms and cognitive reserve. This possible relationship should be considered in order to promote a functional daily performance that promotes delay in the progression to dementia. However, the interaction between cognitive reserve and cognitive function is complex and in our population with MCI none of the aspects regarding cognitive reserve served as a potential predictor of MoCA, paradoxically.

## Figures and Tables

**Table 1:** Demographic and clinical characteristics.

Variable	Male (*n* = 56)	Female (*n* = 69)	Total (*n* = 125)	*p*-Value
Age, mean ± SD	74.04 ± 7.42	74.43 ± 5.98	74.26 ± 6.64	ns
Age group, *n* (%)				
60–74	22 (39.3)	33 (47.8)	55 (44.0)	
75–90	34 (60.7)	36 (52.2)	70 (56.0)	
Study level, *n* (%)				
No study	2 (3.6)	2 (2.9)	4 (3.2)	
Basic	28 (50.0)	39 (56.5)	67 (53.6)	
Primary	11 (19.6)	17 (24.6)	28 (22.4)	
Secondary	7 (12.5)	4 (5.8)	11 (8.8)	
University	8 (14.3)	7 (10.1)	15 (12.0)	
Marital status, *n* (%)				
Single	1 (1.8)	4 (5.8)	5 (4.0)	
Married	51 (91.1)	43 (62.3)	94 (75.2)	
Widowed	4 (7.1)	19 (27.5)	23 (18.4)	
Divorced	-	3 (4.3)	3 (2.4)	
Living status, *n* (%)				
Alone	3 (5.4)	14 (20.3)	17 (13.6)	
Couple	49 (87.5)	43 (62.3)	92 (73.6)	
Daughter/son	-	3 (4.3)	3 (2.4)	
Professional care	1 (1.8)	2 (2.9)	3 (2.4)	
Relatives	3 (5.4)	7 (10.1)	10 (8.0)	
Location, *n* (%)				
Urban	28 (50.0)	33 (47.8)	61 (48.8)	
Intermediate	15 (26.8)	20 (29.0)	35 (28.0)	
Rural	13 (23.2)	16 (23.2)	29 (23.2)	
GDS ^1^, *n* (%)				
3	40 (71.5)	48 (69.6)	88 (70.4)	
4	15 (26.8)	20 (28.9)	35 (28.0)	
5	1 (1.8)	1 (1.4)	2 (1.6)	
Type of MCI ^2^				
MCI	14 (25.0)	21(30.4)	35 (28.0)	
MCI mixed	3 (5.4)	6 (8.7)	9 (7.2)	
MCI vascular	2 (3.6)	3 (4.3)	5 (4.0)	
MCI amnesic /probable AD ^3^	16 (28.6)	19 (27.5)	35 (28.0)	
Others	10 (17.8)	4 (5.7)	14 (11.2)	
Unknown	11(19.6)	16 (23.2)	27 (21.6)	
Geriatric depression Scale, mean ± SD	4.04 ± 3.24	4.93 ± 3.07	4.53 ± 3.16	ns
Geriatric depression Scale group, *n* (%)				
No depression	39 (69.6)	37 (53.6)	76 (60.8)	
Depression probably	12 (21.4)	27 (39.1)	39 (31.2)	
Established depression	5 (8.9)	5 (7.2)	10 (8.0)	
MoCA ^4^, mean ± SD	18.96 ± 4.8	16.81 ± 5.0	17.78 ± 5.01	0.016
CRIq ^5^, mean ± SD				
CRI Index	99.1 ± 19.4	88.3 ± 17.0	93.2 ± 18.9	0.001
CRI-Education	101.7 ± 19.7	98.6 ± 16.2	100.0 ± 17.9	ns
CRI-WorkingActivity	104.1 ± 17.9	86.6 ± 16.4	94.5 ± 19.1	<0.001
CRI-LeisureTime	92.6 ± 21.1	88.5 ± 21.0	90.3 ± 21.0	ns

^1^ GDS: Global Deterioration Scale. ^2^ MCI: Mild Cognitive Impairment. ^3^ Probable AD: Probable Alzheimer Disease. ^4^ MoCA: The Montreal Cognitive Assessment. ^5^ CRIq: Cognitive Reserve Index Questionnaire.

**Table 2:** Mean and standard deviation of MoCA by age, education level and sex in Spanish MCI.

	Male (*n* = 56)			Female (*n* = 69)		
Variable	Basic	Primary	Higher	Basic	Primary	Higher
Age year						
60–74						
*N*	8	7	7	18	9	6
Mean	19.75	19.14	21.00	16.00	17.67	17.50
SD	5.17	7.08	5.38	4.899	5.657	6.80
75–90						
*N*	22	4	8	23	8	5
Mean	17.32	20.00	20.25	15.78	18.63	19.20
SD	3.63	5.03	4.528	4.85	4.17	4.02

**Table 3:** Correlation analyses between MoCA and age, gender and level of education.

Variable	Age	Gender	Level of Education
MoCA ^1^	r = −0.139	r = −0.215	r = 0.290
*p*-value	*p* = 0.123	*p* = 0.016	*p* = 0.001

^1^ MoCA: Montreal Cognitive Assessment.

**Table 4:** Correlation analyses between CRIq score and MoCA scores.

Variable	CRIq ^2^	CRI-Education	CRI-WorkingActivity	CRI-LeisureTime
MoCA ^1^	r = 0.385	r = 0.231	r = 0.237	r = 0.319
95% Confidence Interval	0.21–0.53	0.06–0.39	0.06–0.40	0.15–0.47
*p*-value	*p* < 0.001	*p* = 0.010	*p* = 0.008	*p* < 0.001

^1^ MoCA: Montreal Cognitive Assessment. ^2^ CRIq: Cognitive Reserve Index Questionnaire.

**Table 5:** Semi-partial correlation between CRIq and MoCA score adjusting for sex, age and depression.

	CRIq ^2^	CRI-Education	CRI-WorkingActivity	CRI-LeisureTime
MoCA ^1^	r = 0.319	r = 0.254	r = 0.126	r = 0.298
*p*-value	*p* < 0.001	*p* = 0.005	*p* = 0.165	*p* = 0.001

^1^ MoCA: Montreal Cognitive Assessment. ^2^ CRIq: Cognitive Reserve Index Questionnaire.

**Table 6:** Univariate regression analyses between CRIq and MoCA.

Dependent Variable	Independent Variable	B	Standard Error	T	*p*-Value
MoCA ^1^	CRIq ^2^	0.277	0.215	1.288	0.200
	CRI ^3^-Education	−0.076	0.098	−0.778	0.438
	CRI ^3^-WorkingActivity	−0.100	0.096	−1.045	0.298
	CRI ^3^-LeisureTime	−0.058	0.096	−0.609	0.544

^1^ MoCA: Montreal Cognitive Assessment. ^2^ CRI: Cognitive Reserve Index Questionnaire. ^3^ CRIq: Cognitive Reserve Index.
